# Cement augmentation for treatment of high to mid-thoracic osteoporotic compression fractures, high-viscosity cement percutaneous vertebroplasty versus balloon kyphoplasty

**DOI:** 10.1038/s41598-022-22019-0

**Published:** 2022-11-12

**Authors:** Shiny Chih-Hsuan Wu, An-Jhih Luo, Jen-Chung Liao

**Affiliations:** Department of Orthopedics Surgery, Bone and Joint Research Center, Chang Gung Memorial Hospital, Chang Gung University, No. 5, Fu-Shin Street, Kweishian, Taoyuan, 333 Taiwan

**Keywords:** Health care, Medical research

## Abstract

Whilst the majority of the literature suggests that balloon kyphoplasty (BKP) can relieve pain associated with vertebral compression fractures (VCFs), evidence of high-viscosity cement (HVC) vertebroplasty (VP) or low viscosity cement (LVC) BKP for the treatment of VCFs at the levels of high and mid-thoracic vertebrae remains limited. The purpose of this study was to identify the different outcomes between HVC VP and LVC BKP used to repair high (T4–6) and mid (T7–9)-thoracic VCFs. A total of 114 patients with painful collapsed single-level vertebrae at high to mid-thoracic level who had undergone HVC VP or LVC BKP at a single tertiary medical center was reviewed retrospectively. All patients were divided into the HVC VP group (n = 72) and the LVC BKP group (n = 42). Clinical outcomes including demographic data and visual analogue scale (VAS) were compared. Radiographic data were collected preoperatively, postoperatively, and at final follow-up. More volume (ml) of cement injection was seen in the LVC BKP group (4.40 vs. 3.66, p < 0.001). The operation time (minutes) of the HVC VP group was significantly less than that of the LVC BKP group (33.34 vs. 39.05, p = 0.011). Leakage rate of cement was also fewer in the HVC VP group (26/72 vs. 27/42, p = 0.004). Compared with preoperative data, the VAS was improved after surgery in both groups. The LVC BKP group corrected more middle vertebral body height and local kyphosis angle than the HVC VP group. The outcomes of LVC BKP were not superior to that of HVC VP. HVC VP might be a good alternative to LVC BKP in the treatment of osteoporotic VCFs in high to mid-thoracic spine.

## Introduction

Osteoporosis in the elderly population remains a major public health challenge. Any sudden force of axial compression, distraction, and/or rotation on brittle bone could lead to osteoporotic fractures. By 2025, 3 million osteoporotic fractures and $25 billion in related health care costs will occur in the USA^1^. Vertebral compression fractures (VCFs) account for one-fourth of all osteoporotic-related fractures^1^. VCFs can lead to severe back pain, loss of mobility, spinal deformities, and even neural compromise with neurologic deficits^2,3^. The thoracolumbar junction (T12-L2) accounts for 60–75% of VCFs secondary to osteoporosis, and 30% are involved in the L3–L5 region^4^.

Although conservative treatment including bed rest, analgesics, and bracing can manage most patients with VCFs, debilitating pain and substantial functional limitations often continue to greatly impact their quality of life. Percutaneous cement augmentation for VCFs have acquired popularity in spine surgeons because of their dramatic pain relief and improvement towards life quality for patients^5,6^. These percutaneous cement augmentation procedures can be divided into the following: simple vertebroplasty (VP), balloon kyphoplasty (BKP), and vertebral stenting^7–9^. All these three methods require polymethylmethacrylate (PMMA) to stabilize the fracture and alleviate pain. Most complications from the above procedures are related to the leakage of PMMA cement beyond the fractured vertebral body. While most cases of cement leakage are asymptomatic, they can potentially result in infection, nerve tissue injury, pulmonary emboli, and even death^10^. Burton et al. believed that cement viscosity and injection volume are critical factors for controlling cement leakage^11^. Subsequently, various materials and novel bone cements have been developed and introduced in the market to prevent this condition. Some authors even suggested that cement leakage could be eliminated completely when the viscosity of the material was increased to a consistency that is comparable to dough^12^.

Direct comparisons between high viscosity cement (HVC) VP and low viscosity cement (LVC) BKP have been reported in the literature sporadically^13,14^. The site of injury where treatment occurred in most cases of these studies were at the thoracolumbar junction. The management of high (T4–T6) to mid- (T7–T9) thoracic VCFs has been largely unexplored. To the best of our knowledge, the current studies focusing on the outcomes of HVC VP compared with LVC BKP for high to mid-thoracic VCFs are limited. Thus, the goal of this study was to investigate and compare clinical outcomes using HVC VP and LVC BKP in the treatment of the high to mid-thoracic VCFs.

## Materials and methods

This retrospective study commenced after permission from Chang Gung Memorial Foundation Institutional Review Board (IRB) (number: 202101883B0) was granted. All methods in this study were performed in accordance to relative guidelines and regulations. The written informed consent was waived by Chang Gung Memorial Foundation IRB as this study only involved review of radiographs and medical charts, and did not disclose personal patient information. From January 2015 to December 2020, consecutive series of patients who underwent cement augmentation for spinal lesions at a single tertiary medical center were reviewed. We included patients with single level osteoporotic VCF at levels T4-T9, who received treatment with HVC VP or LVC BKP. Diagnosis was made from plain films, and additionally confirmed with either computed tomography (CT) or magnetic resonance imaging (MRI). Exclusion criteria included patients with high-energy mechanism trauma injury, infection, primary or metastatic malignancy, and those who previously received in situ vertebral-level open reduction and internal fixation.

We recorded patient demographics, comorbidities, the vertebral level of the compression fracture, time of procedure (operation time did not include cement coagulation time), hospital length of stay, complications, and clinical outcome results. Clinical outcomes were evaluated by a pain scale determined by the visual analogue scale (VAS), a continuous scale from 0 to 10, with 0 indicating no discomfort/pain and 10 indicating extreme pain. Patients were asked to rate their pain after a verbal explanation of the scale. The pain scale was recorded preoperatively and 6 months after surgery at the follow-up visit. As there is no universally accepted cut-off point, we used Boonstra et al.’s study as reference in determining a VAS score greater and equal to 3 as cut-off for moderate-to-severe pain^15^

### Radiographic analysis

**Figure 1:**
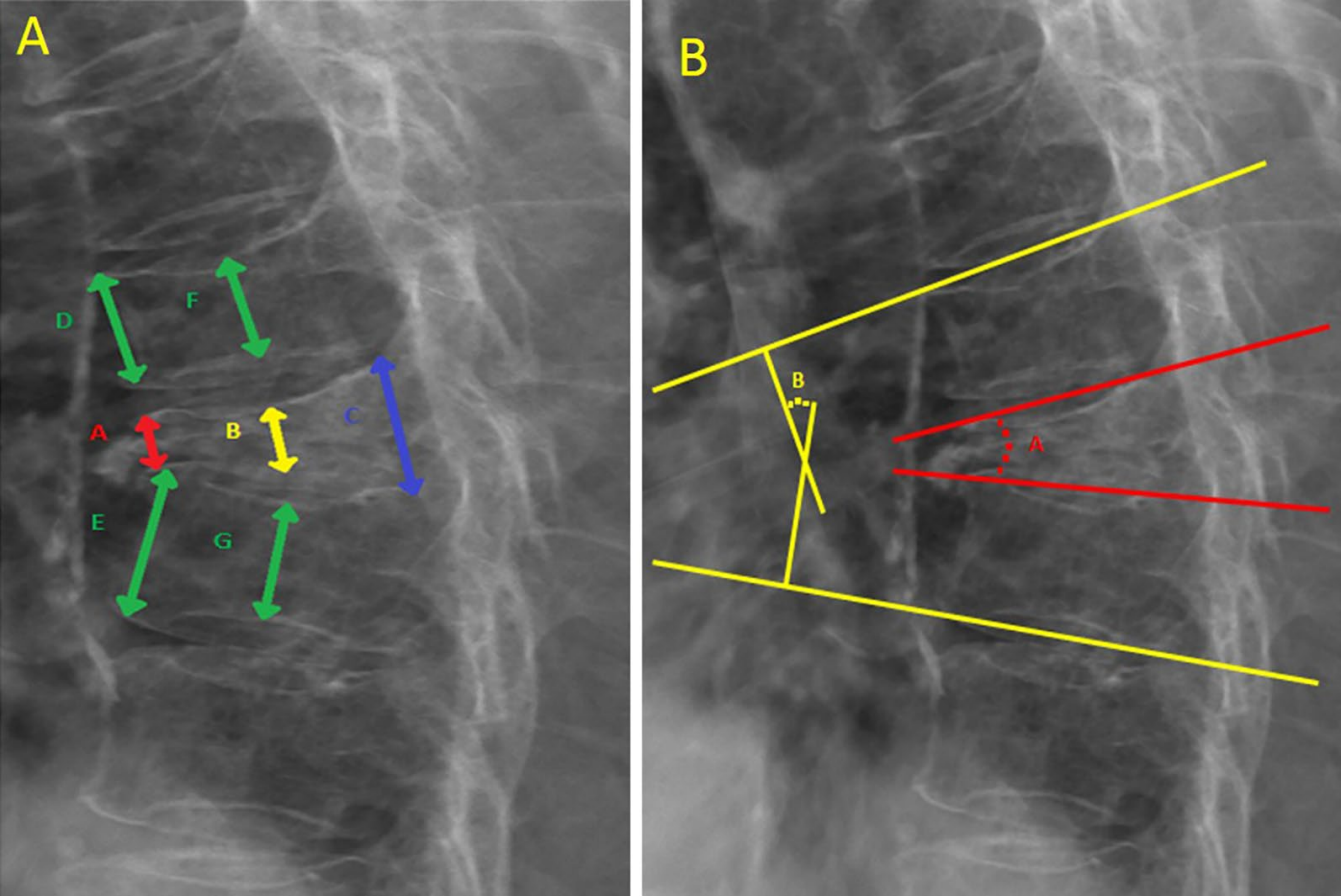
Demonstrated radiographic parameters. (**A**) Evaluation of the severity of compression fracture. A: anterior vertebral height, B: middle vertebral height, C: posterior vertebral height, D: superior anterior vertebral height, E: inferior anterior vertebral height, F: superior middle vertebral height, G: inferior middle vertebral height. (**B**) Measurement of sagittal angle of the fractured vertebrae. A: local kyphotic angle, B: Cobb angle.

Radiographs of the studied cases were blindly reviewed by an observer who did not attend the surgeries. Radiographic parameters included the preoperative, postoperative, and 6-month follow-up anterior vertebral height (AVH), middle vertebral height (MVH), posterior vertebral height (PVH), local kyphotic angle (KA), and Cobb angle (CA). These parameters were measured manually on plain radiographs in lateral and sagittal planes and were calculated using a formula that had been described by Hsu et al.^16^. Figure 1 demonstrates these radiographic parameters. MRI T2-weighted scans were used to determine preoperative vertebral body vacuum cleft and endplate fractures. Cement leakage was assessed using plain film of the thoracic spine at anterior–posterior and lateral views on the day of operation and day 1 postoperatively. Adjacent fractures were evaluated using plain films of the thoracic spine at follow-up.

### Surgical procedures

**Figure 2:**
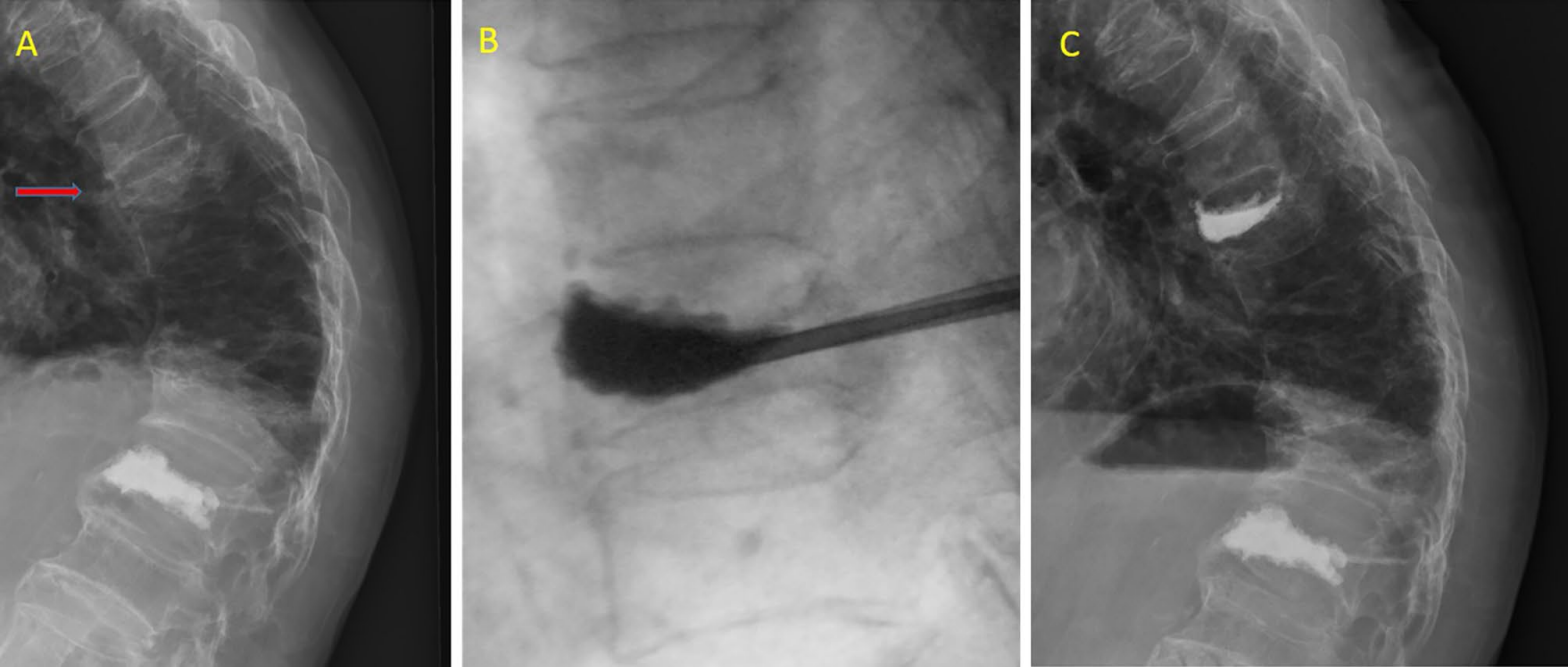
A case of T8 vertebral compression fracture treated with high-viscosity cement vertebroplasty. (**A**) The arrow in the preoperative lateral radiograph points to the fracture level. (**B**) Intraoperative radiograph. (**C**) Postoperative lateral radiograph shows fractured vertebrae filled with high-viscosity cement.

**Figure 3:**
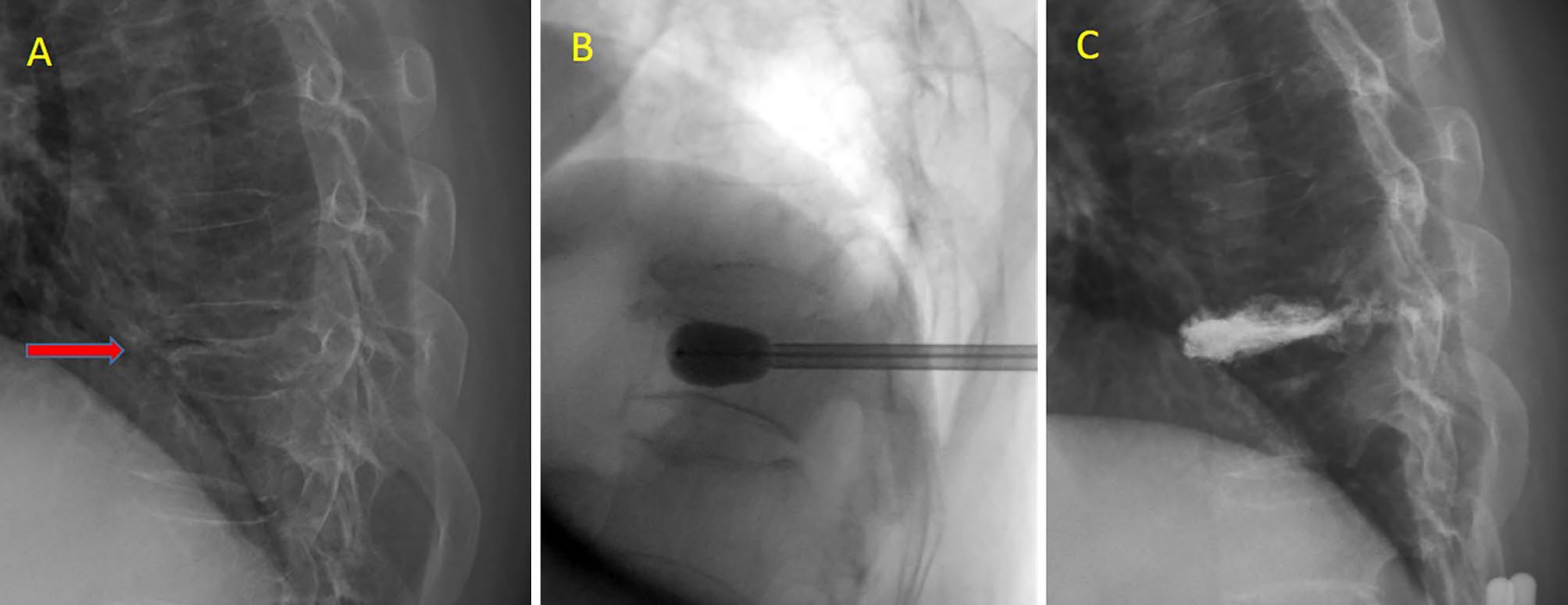
A case of T9 vertebral compression fracture treated with low-viscosity cement balloon kyphoplasty. (**A**) The arrow in the preoperative lateral radiograph points to the fracture level. (**B**) Intraoperative radiograph shows the balloon inflated in the fractured vertebrae. (**C**) Postoperative lateral radiograph demonstrates cement filled in the fractured vertebrae.

The surgical procedures for all patients treated with HVC VP (Confidence Spinal Cement, DePuy Spine, UAS) or LVC BKP (Cement Dispenser System Medinaut-I**,** iMedicom, Korea) were as follows. Patients were placed in a prone position under antiseptic conditions with administration of a dose of prophylactic intravenous antibiotics. During the procedure, all patients were awake under intravenous fentanyl and local lidocaine anesthesia. Under fluoroscopy, a puncture needle was placed percutaneously into the fractured vertebrae through a unilateral or bilateral transpedicular approach. In general, all patients received a unilateral approach. If it was evident that the cement did not project cross the midline on fluroscopy, was then a bilateral approach utilized. The HVC VP procedure was similar to the LVC BKP procedure except for balloon insertion and inflation. The volume of injected cement was dependent on the bone defect and the individual patient's condition. Figure 2 shows a case treated with HVC VP and Fig. 3 demonstrates a case treated with LVC BKP.

### Statistical analysis

The chi-square test and Fisher’s exact test were used for analysis of categorical variables and the independent-sample T-test or analysis of variance (ANOVA) for the analysis of continuous variables. The cut-off for statistical significance was p < 0.05.

### Ethical approval

This study was performed after obtaining Institutional Review Board (IRB) approval of Chang Gung Medical Foundation. IRB No. 202101883B0.

## Results

### Demographic and surgery data

A total of 114 patients with painful collapsed single-level vertebrae at high to mid-thoracic level who underwent vertebral body augmentation were enrolled. According to their surgical procedures, 72 patients were treated with HVC VP versus 42 with LVC BKP. In the HVC VP group, there were 57 women and 15 men (mean age: 74.61 ± 13.41 years). In the LVC BKP group, there were 38 women and 4 men (mean age: 77.57 ± 7.14 yeas). Other demographic data including body mass index, comorbidities, and osteoporosis T-score were similar between the two groups. A falls accident was the cause for 44.4% of the compression fractures in the HVC VP group and 61.9% in the LVC BKP group; the other patients appeared to have sustained vertebral compression fractures for no apparent reason. Evaluation of preoperative MRI data showed no significant differences between the groups for the presence of a vacuum cleft and endplate fractures of the vertebral body. The average hospital length of stay was 3.35 ± 1.41 days and 3.48 ± 2.22 days in the HVC VP and LVC BKP groups, respectively. The average last follow-up time was 1 year.

The volume of cement injected was significantly higher in the LVC BKP group (4.40 ml ± 1.71 ml) than in the HVC VP group (3.66 ml ± 1.36 ml) (p < 0.001). However, the HVC VP group was associated with fewer cases of cement leakage (26/72 vs. 27/42, p = 0.004). There were no neurologic deficits or symptomatic emboli in these patients. The operation time for the HVC VP group (33.34 ± 6.02 min) was significantly less than that for the LVC BKP group (39.05 ± 10.11 min) (p = 0.011). The operation time did not include cement coagulation time. During the 6 month follow-up period, there was no difference between the groups for the incidence of adjacent fractures following cement augmentation (6/72 vs. 3/42, p = 0.445). Pain relief following surgery was highly desired by patients. Both groups showed considerable improvement in the follow-up VAS scores (LVC BKP: 5.60 to 2.17; HVC VP: 5.86 to 2.40). There were no significant differences in the preoperative and postoperative VAS scores between the two groups. Table 1 summarizes the demographic and clinical results.

**Table 1:** Patient demographic data: LVC BKP versus HVC VP.

Variables	LVC BKP (N = 42)	HVC VP (N = 72)	*p-value*
**Basic data**			
Male/Female (N, ratio)	4/38	15/57	0.192
Age (years, mean ± SD)	77.57 ± 7.14	74.61 ± 13.41	0.127
Body mass index (kg/m^2^, mean ± SD)	25.65 ± 4.13	23.53 ± 3.68	0.005*
**Levels**			
T4–T6 (N)	7	17	0.380
T4 (N)	0	2	
T5 (N)	5	2	
T6 (N)	2	13	
T7–T9 (N)	35	55	
T7 (N)	12	18	
T8 (N)	12	22	
T9 (N)	11	15	
BMD (g/cm^2^)	− 3.16 ± 1.46	− 2.87 ± 1.48	0.116
Osteoporosis (T score < − 2.5) (N)	30	45	0.226
Falls injury (N)	26	32	0.361
**Hospital course**			
Hospital length of stay (days, mean ± SD)	3.48 ± 2.22	3.35 ± 1.41	0.063
Pre-OP VAS (mean ± SD)	5.60 ± 1.53	5.86 ± 1.03	0.320
F/U VAS (mean ± SD)	2.17 ± 0.49	2.40 ± 1.53	0.229
F/U VAS ≥ 3 (N)	9	22	0.291
**Surgery related variables**			
Vacuum cleft (N)	21	35	0.886
Endplate fracture (N)	36	67	0.200
Uni-pedicle/bipedicle (N, ratio)	42/0	63/9	0.025*
Left/right (N, ratio)	42/0	58/5	0.082
Cement volume (ml, mean ± SD)	4.40 ± 1.71	3.66 ± 1.36	< 0.001*
Operation time (min, mean ± SD)	39.05 ± 10.11	33.34 ± 6.02	0.011
Cement leakage (N)	27	26	0.004*
Adjacent fracture (N)	3	6	0.445

*LVC BKP* low-viscosity cement balloon kyphoplasty, *HVC VP* high-viscosity cement vertebroplasty, *N* number, *SD* standard deviation, *kg* kilogram, *m* meter, *min* minutes, *g* gram, *cm* centimeter, *BMD* bone mass density, *VAS* visual analog scale, *Pre-OP* pre-operative, *Post-OP* post-operative, *F/U* follow-up.

*p value < 0.05.

### Radiographic outcomes

Both groups showed increased AVH, MVH, PVH, corrected KA, and CA after the cement augmentation procedure. Before surgery, both groups had similar AVH, MVH, PVH, KA, and CA data. After surgery, the LVC BKP group obtained significantly greater height (mm) in postoperative AVH and MVH (16.05 mm ± 3.20 mm vs. 14.52 mm ± 4.52 mm, p = 0.043; 12.17 mm ± 2.77 mm vs. 10.42 mm ± 4.17 mm, p = 0.010), but there was no significant difference for PVH between the two groups after surgery. The LVC BKP group had a significantly more corrected MVH than the HVC VP group (ΔMVH: 2.59 mm ± 2.38 mm vs. 1.25 mm ± 3.15 mm, p = 0.020). At final follow up, both groups again showed similar AVH, MVH, and PVH. Recurrent vertebral height loss following cement augmentation was encountered in both groups, but there was no significant difference between the groups (percentage of vertebral body height change during follow-up: ΔAVH: -6.99% ± 6.30% vs. -5.83% ± 6.90%, p = 0.441; ΔMVH: -7.40% ± 7.36% vs. -7.84% ± 3.09%, p = 0.817). The values of local angles and vertebral body–associated parameters are summarized in Table 2.

**Table 2:** Radiographic data between LVC BKP and HVC VP.

Variables	LVC BKP (N = 42)	HVC VP (N = 72)	*p-value*
Vertebral width (mm, mean ± SD)	30.32 ± 2.62	30.67 ± 3.37	0.557
Superior reference of AVH (mm, mean ± SD)	19.25 ± 3.89	18.98 ± 4.43	0.745
Inferior reference AVH (mm, mean ± SD)	19.67 ± 5.43	20.33 ± 4.12	0.467
Superior reference of MVH (mm, mean ± SD)	15.34 ± 3.25	15.39 ± 4.47	0.947
Inferior reference MVH (mm, mean ± SD)	15.68 ± 4.49	16.82 ± 3.84	0.153
**Vertebral body height**			
Pre-OP AVH (mm, mean ± SD)	13.23 ± 3.87	12.38 ± 5.18	0.326
Post-OP AVH (mm, mean ± SD)	16.05 ± 3.20	14.52 ± 4.52	0.043*
∆ AVH (mm, mean ± SD)	2.82 ± 2.82	2.19 ± 2.60	0.238
F/U AVH (mm, mean ± SD)	14.82 ± 4.23	14.16 ± 4.26	0.413
F/U ∆ AVH (to Post-OP) (mm, mean ± SD)	− 1.09 ± 0.99	− 0.93 ± 1.20	0.525
Pre-OP MVH (mm, mean ± SD)	9.59 ± 3.33	9.19 ± 4.64	0.596
Post-OP MVH (mm, mean ± SD)	12.17 ± 2.77	10.42 ± 4.13	0.010*
∆ MVH (mm, mean ± SD)	2.59 ± 2.38	1.25 ± 3.15	0.020*
F/U MVH (mm, mean ± SD)	11.17 ± 2.62	10.11 ± 3.73	0.133
F/U ∆ MVH (to Post-OP) (mm, mean ± SD)	− 0.92 ± 0.98	− 0.89 ± 1.10	0.919
Pre-OP PVH (mm, mean ± SD)	18.80 ± 2.76	18.77 ± 3.21	0.960
Post-OP PVH (mm, mean ± SD)	20.06 ± 2.59	19.57 ± 2.95	0.376
∆ PVH (mm, mean ± SD)	1.27 ± 1.68	0.89 ± 1.37	0.203
F/U PVH (mm, mean ± SD)	19.37 ± 2.47	19.38 ± 2.81	0.979
F/U ∆ PVH (to Post-OP) (mm, mean ± SD)	− 0.49 ± 0.63	− 0.66 ± 0.88	0.301
% of pre-op vertebral compression ratio (%, mean ± SD)	30.40 ± 14.52	35.80 ± 19.86	0.098
% of pre-op AVH compression (%, mean ± SD)	28.58 ± 29.56	34.91 ± 30.68	0.284
% of pre-op MVH compression (%, mean ± SD)	35.16 ± 27.88	38.39 ± 38.84	0.609
% of post-op AVH compression (%, mean ± SD)	13.63 ± 27.80	23.29 ± 29.22	0.092
% of post-op MVH compression (%, mean ± SD)	16.92 ± 32.05	31.66 ± 33.42	0.026*
% of F/U AVH compression (%, mean ± SD)	21.64 ± 22.45	24.83 ± 26.93	0.566
% of F/U MVH compression (%, mean ± SD)	24.67 ± 29.52	33.80 ± 27.83	0.152
% of ∆ AVH compression after operation (%, mean ± SD)	14.95 ± 15.19	11.47 ± 14.95	0.244
% of ∆ MVH compression after operation (%, mean ± SD)	14.25 ± 14.50	6.31 ± 27.85	0.092
% of F/U ∆ AVH (to Post-OP) (%, mean ± SD)	− 6.99 ± 6.33	− 5.83 ± 6.90	0.441
% of F/U ∆ MVH (to Post-OP) (%, mean ± SD)	− 7.40 ± 7.36	− 7.84 ± 9.09	0.817
**Kyphotic angle**			
Pre-op Kyphotic angle (degree, mean ± SD)	13.74 ± 5.73	15.06 ± 6.73	0.287
Post-op Kyphotic angle (degree, mean ± SD)	8.13 ± 4.75	9.72 ± 6.32	0.167
∆ Kyphotic angle (degree, mean ± SD)	− 5.61 ± 4.09	− 5.46 ± 4.58	0.864
% of ∆ Kyphotic angle (%, mean ± SD)	41.24 ± 24.81	36.98 ± 24.99	0.390
**Cobb angle**			
Pre-op Cobb angle (degree, mean ± SD)	20.17 ± 11.12	20.52 ± 9.90	0.863
Post-op Cobb angle (degree, mean ± SD)	13.95 ± 9.27	16.46 ± 9.74	0.187
∆ Cobb angle (degree, mean ± SD)	− 6.23 ± 5.29	− 4.22 ± 4.23	0.032*

*LVC BKP* low-viscosity cement balloon kyphoplasty, *HVC VP* high-viscosity cement vertebroplasty, *N* number, *mm* millimeter, *SD* standard deviation, *Pre-OP* pre-operative, *Post-OP* post-operative, *F/U* follow-up, *AVH* anterior vertebral body height, *MVH* middle vertebral body height, *PVH* posterior vertebral body height, % percentage.

*p value < 0.05.

## Discussion

VP and BKP are common interventions for the treatment of osteoporotic VCFs. The difference between VP and BKP is that BKP adds a procedure of balloon inflation in the collapsed vertebrae. Both interventions use bone cement to stabilize the fracture. Clinical studies of different cement augmentation procedures have been encouraging. Several studies have suggested both VP and BKP improve quality of life, pain relief, functionality, and restores vertebral body height^10^. Zhao et al. performed a meta-analysis study which demonstrated patients treated with BKP was more effective than VP according to superior scores for long term VAS and Oswestry Disability Index, improved KA and mean vertebral body height, and significantly reduced risk of cement leakage^17^. However, some studies did not support that BKP is superior to VP for pain relief and functional improvement^18,19^. In this current study, VAS outcomes reflecting pain relief after vertebral body augmentation at high to mid-thoracic vertebrae was similar for  HVC VP and LVC BKP.

The most frequent area involved in VCFs is the thoracolumbar junction (T12-L2), followed by the lower lumbar area (L3–L5)^20^. By contrast, the incidence of VCFs at high (T4–T6) and mid- (T7–T9) thoracic vertebrae is lower and associated research is limited. The thoracic spine is part of the thoracic cage, which serves to protect vital organs and provide rigid support to bear and disperse substantial axial loading forces. Patients with a compression fracture over the high to mid-thoracic vertebrae can suffer high to mid-back pain, which can be exacerbated with respiration and may radiate to the anterior chest. Anatomically, the thoracic vertebrae is characterized by small pedicles with the diameter becoming narrower as the thoracic level gets higher. Therefore, nervous tissue would likely be intolerable when cement leakage into the thoracic canal occurs. To minimise the risk of cement leakage, Liu et al. recommended that LVC BKP is preferred over LVC VP for osteoporotic VCFs of mid-thoracic vertebrae^21^. Another key factor to reduce the incidence of cement leakage is viscosity of the cement in VP. Alhashash et al. demonstrated that HVC VP had a relatively low risk of cement leakage for patients with VCFs^22^. However, they focused mostly on lower thoracic and lumbar spine levels. Based on our study, the cement leakage rate is lower with HVC VP in relation to LVC BKP (36% vs. 64%, p = 0.004) after cement augmentation for high to mid-thoracic vertebrae. A meta-analysis study by Chen et al. also confirmed that HVC VP holds the lowest rate for cement leakage after cement augmentation when compared to LVC BKP and LVC VP^23^.

Cement injection volume has been reported as one of the risk factors for cement leakage in percutaneous VP and BKP. Zhu et al. recommended that < 3.5 mL of bone cement per vertebrae should be injected to minimize the risk of volume-associated cement leakage^24^. Similar results are also observed in BKP with Chen et al. demonstrating decreased volume of cement injection could also reduce the incidence of cement leakage^25^. Although most leaks were clinically asymptomatic, they carry the risk of pulmonary embolism and neurologic compression which are considered major complications in cement augmentation procedures. In this study, cement volume of injection was significantly lower in the HVC VP group (3.66 ml vs. 4.40 ml, p < 0.001). Moreover, HCV VP alleviates the need for bone cavity creation within the injured vertebra, thus significantly reducing the procedure time (31 min vs 39 min, p = 0.011), Thus, we believe HVC VP is more reliable than LVC BKP when applied to high to mid-thoracic vertebrae.

The biomechanics of the fractured segment are altered following cement augmentation. The reconstructed vertebrae is more rigid than its adjacent segments. It acts as an upright pillar that reduces the inward physiologic bulging of the endplates of the augmented vertebrae. Liao et al. made a finite element model of osteoporotic VCFs with cement augmentation with VP, BKP, and vertebral stents^26^. The results showed all these procedures would increase stress on the endplates of the adjacent segments, especially the superior levels during flexion. Another finite element study revealed that when cement filling volume reached 30–40.5% of the volume of a vertebral body, this also increases the stress tolerated by the adjacent segments. However, when the injected cement volume exceeded the defined range, stress distributions on fractured and adjacent vertebral bodies not only increased but led to development of adjacent vertebral fractures^27^. Clinically, the incidence of adjacent fractures have been reported to range from 5.5 to 52% after VP and BKP^17,28,29^. In our study, the incidence of adjacent fractures following HVC VP and LVC BKP was 8.3% and 9.1%, respectively. We believed the incidence of adjacent fractures at high to mid-thoracic vertebral levels would be far less than that at the thoracolumbar junction because a smaller vertebral body size has consequently less cement injection and additional protection is encouraged by the thoracic cage.

In this study, all radiographic results including AVH, MVH, PVH, local kyphotic angle, and Cobb angle showed significant postoperative improvement in both groups. The HVC VP group showed comparable radiographic outcomes to those of the LVC BKP group in terms of kyphotic reduction, but with less vertebral body height restoration (determined by postoperative AVH and MVH). It is believed that the effect of balloon inflation on the injured vertebrae and more cement injected in the LVC BKP group resulted in these radiographic advantages immediately after surgery. However, more severe re-collapsed vertebrae were also shown in the LVC BKP group, which resulted in final follow-up radiographic parameters that had no statistical difference between two groups. These changes in radiographic data did not influence clinical outcomes.

In the past two decades, the majority of the literature published discuss the differences LVC VP and LVC BKP for spinal compression fractures. However, studies that compare HVC VP and LVC BKP is very scarce. After a search in PubMed, we found only four clinical research studies published discussing the use of HVC VP versus LVC BKP in the treatment of osteoporotic VCFs^13,14,30,31^. Dr. Georgy was the first author to describe his experiences in using HVC VP and BKP for osteoporotic VCFs, and his data showed HVC VP had a significantly lower rate of cement leakage^30^. Data from Wang et al. revealed injected volume was higher with BKP but cement leakage rate was lower in HVC VP^14^. Sun et al. and Lin et al. all demonstrated similar clinical results for VAS and Oswestry Disability Index (ODI) scores with either HVC VP or BKP, and restoration of height for the middle vertebrae appeared superior in the BKP group^13,31^. The biggest difference between our study and the above four reference papers was that our study included cases that involved only the high to mid-thoracic spine, but the thoracolumbar junction stood the majority of cases in the reference articles. Nevertheless, our data drew similar results to that of the above references.

Indeed, there are limitations to our study. First, the nature of a retrospective study might include inherent bias. Moreover, clinical outcomes for daily function such as ODI or 36-Item Short Form Survery (SF-36) would provide additional beneficial clinical functional information but could not be evaluated due to unavailability of data in a retrospective study. Second, the procedures were performed by different surgeons at our center, and we were unable to account for technique variation. Third, although the results were clear and comparable between the groups, a longer follow-up period is necessary to assess whether HVC can alleviate BKP-related risks in the treatment of osteoporotic VCFs in high to mid-thoracic vertebrae. Fourthly, the medical insurance policy in Taiwan does not subsidize the use of CT or MRI for evaluating bone cement leakage or adjacent fractures respectively, therefore the diagnosis using plain film inherently has a high false negative incidence and data may therefore be restricted.

## Conclusions

In this study, both HVC VP and LVC BKP groups were safe and effectively achieved pain relief for patients with high to mid-thoracic osteoporotic VCFs. The mean operation time of HVC VP was significantly shorter than that of LVC BKP, but vertebral body height and local kyphosis angle could be corrected better postoperatively by LVC BKP. In addition, HVC VP demonstrated a lower bone cement leakage rate and incidence of adjacent segment fracture compared to LVC BKP. Thus, HVC VP might be a good alternative to LVC BKP in the treatment of osteoporotic VCFs in high to mid-thoracic spine.

## Data Availability

The data used to support the finding of this study are available from the corresponding author upon request.
